# Supplementary material for: Mismatch Repair Status in Patient-Derived Colorectal Cancer Organoids Does Not Affect Intrinsic Tumor Cell Sensitivity to Systemic Therapy
Source: Cancers (Basel). 2021 Oct 29;13(21):5434. doi: 10.3390/cancers13215434 (PMC8582471; doi:10.3390/cancers13215434)
Supplement: Supplementary file 1 [file cancers-13-05434-s001.zip › cancers-1417960-supplementary.pdf]

Supplementary

Mismatch repair status in patient-derived colorectal cancer organoids does not affect intrinsic tumor cell sensitivity to systemic therapy

Emre Küçükköse, G. Emerens Wensink, Celine M. Roelse, Susanne J. van Schelven, Daniëlle A.E. Raats, Sylvia F. Boj, Miriam Koopman, Jamila Laoukili, Jeanine M.L. Roodhart and Onno Kranenburg

Table S1. Composition of organoid culture medium

Component	Source (catalag number)	Concentration
**Advanced (DMEM/F12) medium**	Gibco (12634-010)	1x (500 mL total)
**HEPES Buffer**	Lonza (17737E)	10 mM
**Penicillin/Streptomycin**	Gibco (15070-063)	50 U/ml
**GlutaMAX**	Gibco (35050-038)	2 mM
**R-Spondin conditioned medium**	293T-HA-RspoI-F cell line	20%
**Noggin conditioned medium**	293T-mNoggin-Fc cell line	100 ng/ml
**B27**	Invitrogen (17504-044)	1x
**Nicotinamide**	Sigma-Aldrich (N0636)	10 mM
**Prostaglandin E2**	Tocris (2296-10)	10 nM
**Gastrin**	Sigma-Aldrich (G9145)	10 nM
**N-acetylcysteine (NAC)**	Sigma-Aldrich (A9165)	1 mM
**A83-01**	SignalChem (A09-900-05)	500 nM
**Human recombinant EGF**	Sigma Aldrich (A9165)	50 ng/mL
**SB202190**	Gentaur (A1632)	10 µM

The table describes the composition of the organoid culture medium. Abbreviations: Dulbecco's Modified Eagle Medium/Ham's F-12 (DMEM/F12), epidermal growth factor (EGF).

Table S2. Overview of chemotherapies and targeted treatments used in drug screens

Drug	Target	Patient C_{MAX}	Chosen concentration range			
**5-FU**	DNA synthesis	0.88 μM[1]	1.5	-	300	μM
**Oxaliplatin**	DNA-alkalyting agent	4.96 μM[1]	0.5	-	200	μM
**SN-38 (irinotecan)**	Topoisomerase I	0.014-0.143 μM[1]	0.0002	-	0.1	μM
**Binimetinib**	MEK	1.0-1.2 μM[2]	0.001	-	15	μM
**Cetuximab**	EGFR	205 μg/ml[1]	10	-	150	μg/ml
**Encorafenib**	BRAF	1.12-4.17 μM[2]	0.01	-	50	μM
**Puromycin**	Protein synthesis	-			20	μg/ml

The table indicates the agents and concentration range used in drug screen assays. As well, the published patient Cmax values are also indicated with the appropriate references.

References:

1. Liston, D. R. & Davis, M. Clinically relevant concentrations of anticancer drugs: A guide for nonclinical studies. Clin. Cancer Res. 23, 3489–3498 (2017);

2. Assessment report Mektovi (binimetinib). European Medicine Agency (2018). Available at: https://www.ema.europa.eu/en/medicines/human/EPAR/mektovi;

3. Van Geel, R. M. J. M. et al. A phase Ib dose-escalation study of encorafenib and cetuximab with or without alpelisib in metastatic BRAF-mutant colorectal cancer. Cancer Discov. 7, 610–619 (2017).

Table S3. Overview drug response AUCs per organoid and agent.

Organoid ID	BRAF status	KRAS status	Agent	AUC
**dMMR1**	BRAF-m (V600E)	WT	5-FU	2,12
**dMMR2**	WT	WT	5-FU	1,71
**dMMR3**	WT	KRAS-m (A146T)	5-FU	1,93
**dMMR4**	BRAF-m (V600E)	KRAS-m (A146T)	5-FU	1,87
**dMMR5**	WT	WT	5-FU	1,88
**dMMR6**	BRAF-m (V600E)	WT	5-FU	2,29
**pMMR1**	BRAF-m	WT	5-FU	3,08
**pMMR2**	WT	KRAS-m	5-FU	2,09
**pMMR3**	WT	WT	5-FU	1,91
**pMMR4**	WT	KRAS-m	5-FU	2,09
**pMMR5**	WT	KRAS-m	5-FU	2,00
**pMMR6**	WT	WT	5-FU	1,76
**dMMR1**	BRAF-m (V600E)	WT	Binimetinib	2,24
**dMMR2**	WT	WT	Binimetinib	3,16
**dMMR3**	WT	KRAS-m (A146T)	Binimetinib	2,93
**dMMR4**	BRAF-m (V600E)	KRAS-m (A146T)	Binimetinib	3,18
**dMMR5**	WT	WT	Binimetinib	3,66
**dMMR6**	BRAF-m (V600E)	WT	Binimetinib	3,39
**pMMR1**	BRAF-m	WT	Binimetinib	3,09
**pMMR2**	WT	KRAS-m	Binimetinib	3,13
**pMMR3**	WT	WT	Binimetinib	3,97
**pMMR4**	WT	KRAS-m	Binimetinib	3,39
**pMMR5**	WT	KRAS-m	Binimetinib	2,10
**pMMR6**	WT	WT	Binimetinib	3,98
**dMMR1**	BRAF-m (V600E)	WT	Cetuximab	1,22
**dMMR2**	WT	WT	Cetuximab	1,04
**dMMR3**	WT	KRAS-m (A146T)	Cetuximab	1,25
**dMMR4**	BRAF-m (V600E)	KRAS-m (A146T)	Cetuximab	1,59
**dMMR5**	WT	WT	Cetuximab	1,44
**dMMR6**	BRAF-m (V600E)	WT	Cetuximab	1,58
**pMMR1**	BRAF-m	WT	Cetuximab	1,42
**pMMR2**	WT	KRAS-m	Cetuximab	1,40
**pMMR3**	WT	WT	Cetuximab	1,41
**pMMR4**	WT	KRAS-m	Cetuximab	1,39
**pMMR5**	WT	KRAS-m	Cetuximab	1,50
**pMMR6**	WT	WT	Cetuximab	1,17
**dMMR1**	BRAF-m (V600E)	WT	Encorafenib	0,96
**dMMR2**	WT	WT	Encorafenib	3,04
**dMMR3**	WT	KRAS-m (A146T)	Encorafenib	3,08
**dMMR4**	BRAF-m (V600E)	KRAS-m (A146T)	Encorafenib	2,48
**dMMR5**	WT	WT	Encorafenib	3,06

**dMMR6**	BRAF-m (V600E)	WT	Encorafenib	2,01
**pMMR1**	BRAF-m	WT	Encorafenib	2,35
**pMMR2**	WT	KRAS-m	Encorafenib	2,68
**pMMR3**	WT	WT	Encorafenib	3,02
**pMMR4**	WT	KRAS-m	Encorafenib	3,19
**pMMR5**	WT	KRAS-m	Encorafenib	3,18
**pMMR6**	WT	WT	Encorafenib	3,11
**dMMR1**	BRAF-m (V600E)	WT	Oxaliplatin	1,79
**dMMR2**	WT	WT	Oxaliplatin	1,82
**dMMR3**	WT	KRAS-m (A146T)	Oxaliplatin	1,80
**dMMR4**	BRAF-m (V600E)	KRAS-m (A146T)	Oxaliplatin	2,24
**dMMR5**	WT	WT	Oxaliplatin	1,51
**dMMR6**	BRAF-m (V600E)	WT	Oxaliplatin	2,10
**pMMR1**	BRAF-m	WT	Oxaliplatin	2,13
**pMMR2**	WT	KRAS-m	Oxaliplatin	2,16
**pMMR3**	WT	WT	Oxaliplatin	1,79
**pMMR4**	WT	KRAS-m	Oxaliplatin	2,34
**pMMR5**	WT	KRAS-m	Oxaliplatin	2,25
**pMMR6**	WT	WT	Oxaliplatin	1,59
**dMMR1**	BRAF-m (V600E)	WT	SN-38	2,32
**dMMR2**	WT	WT	SN-38	2,14
**dMMR3**	WT	KRAS-m (A146T)	SN-38	2,53
**dMMR4**	BRAF-m (V600E)	KRAS-m (A146T)	SN-38	2,42
**dMMR5**	WT	WT	SN-38	1,85
**dMMR6**	BRAF-m (V600E)	WT	SN-38	1,57
**pMMR1**	BRAF-m	WT	SN-38	2,20
**pMMR2**	WT	KRAS-m	SN-38	2,72
**pMMR3**	WT	WT	SN-38	2,30
**pMMR4**	WT	KRAS-m	SN-38	2,34
**pMMR5**	WT	KRAS-m	SN-38	2,21
**pMMR6**	WT	WT	SN-38	2,33

Abbreviations: 5-FU (5-fluorouracil), AUC (area under the curve, obtained using Simpson's rules), BRAF-m (BRAF-mutant), KRAS-m (KRAS-mutant), WT (BRAF & KRAS wildtype).

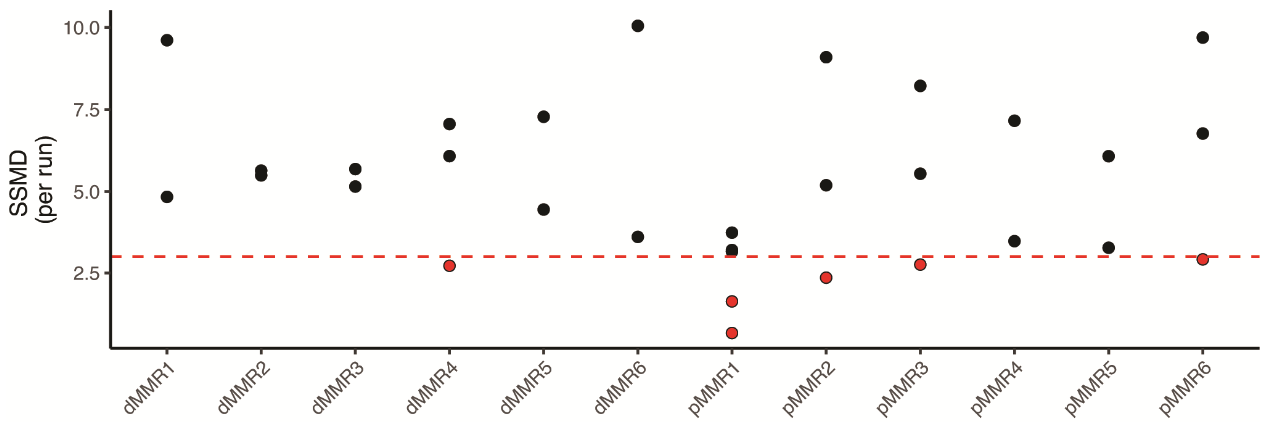

Figure S1: Quality control parameters of drug screens. The Strictly Standardized Mean Difference (SSMD) values are plotted for each analyzed run for a given organoid line. Organoid drug screen runs which were excluded from the analysis (due to SSMD <=3.0) are indicated with a red point.

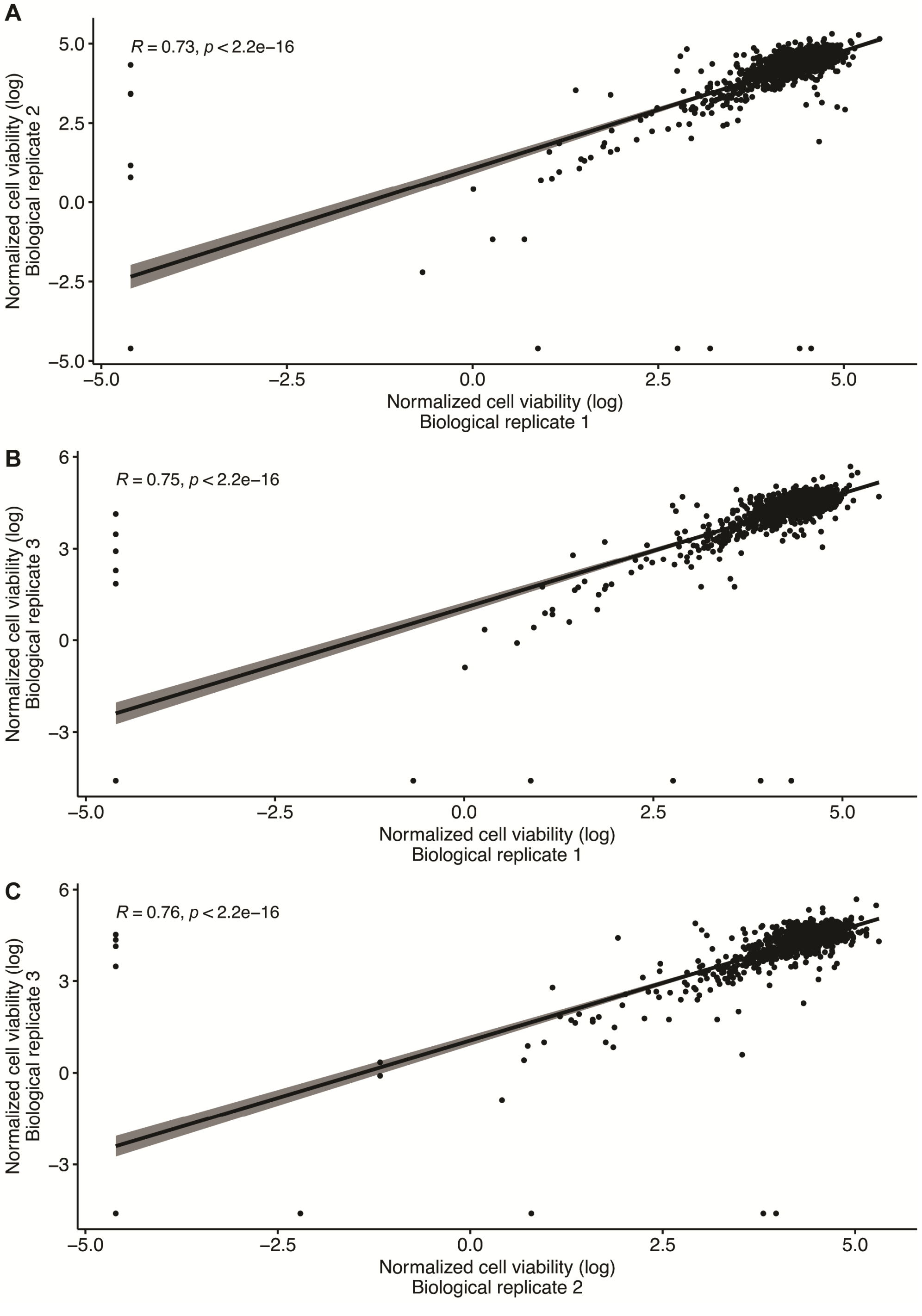

Figure S2: Correlation of biological replicates of normalized cell viability. Normalized cell viability values (log) for all analyzed drug screens are plotted for each biological and technical replicate and the Pearson's R correlation values are displayed. A) Biological replicate 1 versus 2; B) Biological replicate 1 versus 3; C) Biological replicate 2 versus 3.

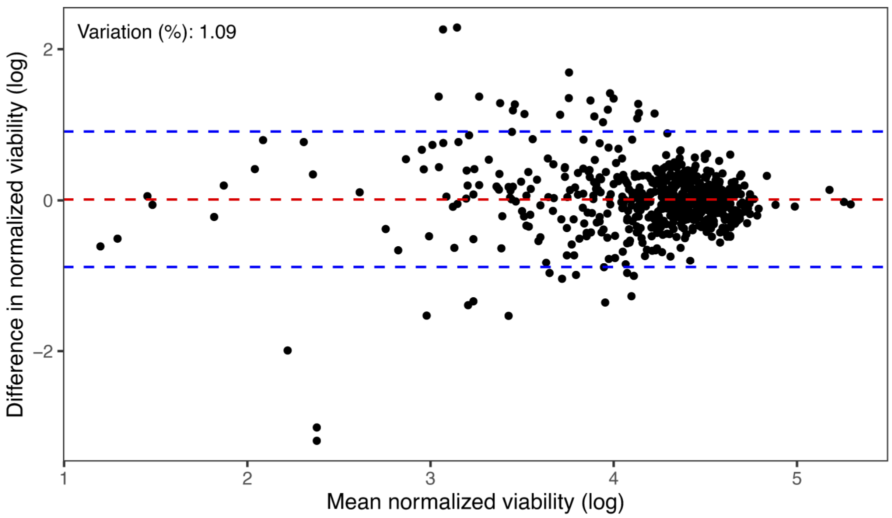

Parameter	Value	95% CI
Bias (Difference)	0.01	-0.02; 0.05
Lower Limit of Agreement	-0.89	-0.95; -0.83
Upper Limit of Agreement	0.91	0.85; 0.97

Figure S3: Difference between duplicate assays. A Bland-Altman plot of the mean normalized viability of the replicates (log) versus the difference in normalized viability between replicates (log) is displayed. The red line indicates the average difference observed between the replicates and the blue lines are the standard deviation of the differences.

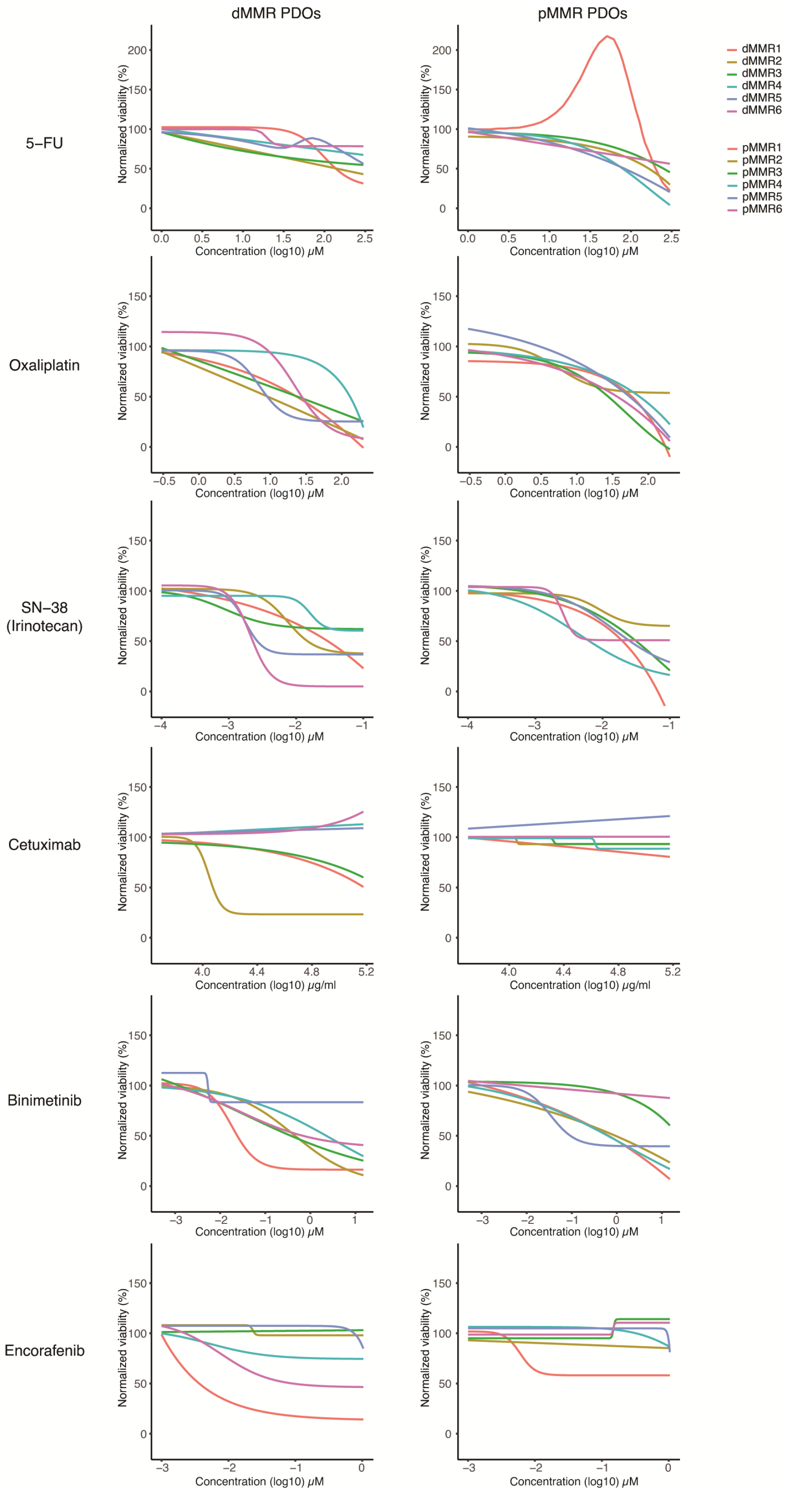

Figure S4: Drug response curves for each systemic therapy agent (dMMR vs pMMR PDOs). An overlay of the individual drug response curves (DRC) for each organoid are displayed. Two side-by-side plots were created per treatment type: dMMR PDOs versus pMMR PDOs.

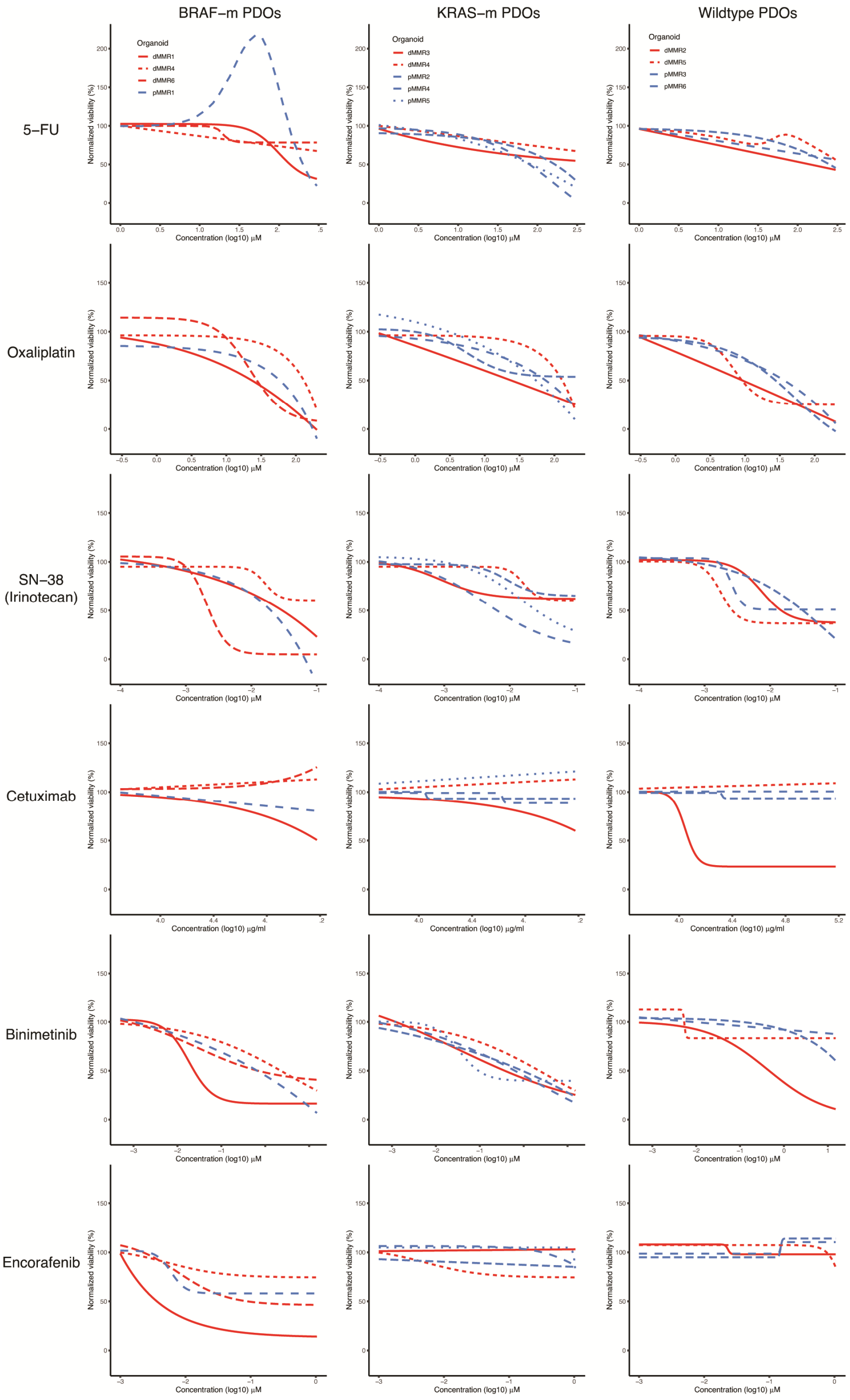

Figure S5: Drug response curves for each systemic therapy agent (BRAF-mutant versus KRAS-mutant versus Wildtype PDOs). An overlay of the individual drug response curves (DRC) for each organoid are displayed, for run 2. Two side-by-side plots were created per treatment type: BRAF-mutant (BRAF-m) in dark red color, KRAS-mutant (KRAS-m) in orange versus BRAF & KRAS wildtype (Wildtype) PDOs in blue.
